# Fifty-millimeter abscess in the ileum caused by perforation from anisakiasis successfully treated with conservative therapy without drainage

**DOI:** 10.1093/omcr/omab033

**Published:** 2021-06-18

**Authors:** Koki Kawanishi, Yoshifumi Ikeda, Masahiko Furotani, Sayaka Tsuboi, Takayuki Kanno, Toru Niwa, Tsunehiro Nagaoka, Yoshinari Tabata, Masayuki Kitano

**Affiliations:** 1 Department of Gastroenterology, Nate Hospital, Kinokawa City, Wakayama, Japan; 2 Department of General Surgery, Wakayama Seikyo Hospital, Wakayama City, Wakayama, Japan; 3 Second Department of Internal Medicine, Wakayama Medical University, Wakayama City, Wakayama, Japan

## Abstract

Intestinal anisakiasis is not only a rare but also a difficult to diagnose parasitic disease. The symptoms are not specific and are often severe and abrupt; therefore, patients are sometimes diagnosed as having surgical abdomen. The clinical imaging findings are remarkable, including ascites, enteritis, ileus, eosinophilic granuloma and sometimes perforation. We experienced a case of intestinal anisakiasis diagnosed on the basis of the *Anisakis*-specific immunoglobulin A level from paired sera and treated successfully with conservative therapy, although ileum perforation was complicated by a 50-mm abscess. Even the large abscess could be treated without drainage in thiscase.

## INTRODUCTION

Anisakiasis is a parasitic disease in humans caused by the incidental ingestion of *Anisakis* larvae, which are present in fresh fish and squid [1]. When the larvae stick to the gastrointestinal membrane and cause various kinds of symptoms, the status is termed as anisakiasis. According to the location where the *Anisakis* larvae are stuck to the human body, anisakiasis is categorized into gastric, intestinal and ectopic anisakiasis [2, 3]. In a series of 15 715 cases of anisakiasis reported in Japan, the proportions of the categories were as follows: gastric anisakiasis, 95.6%; intestinal anisakiasis, 4.1%; and ectopic anisakiasis, 0.3% [4].

The clinical imaging findings in intestinal anisakiasis are remarkable, including ascites, enteritis, ileus, eosinophilic granuloma and sometimes perforation [5]. Patients with intestinal perforations or strangulation normally require surgical therapy, but conservative therapy can be the treatment option in anisakiasis [3, 6]. Cases of nongastric anisakiasis are not only rare but also difficult to diagnose because the small intestine is unreachable by a regular endoscope, although detecting the whole worm visually is the hallmark of the diagnostic procedure [3, 5]. As a result, patients with intestinal anisakiasis diagnosed as acute abdomen or intestinal obstructions might undergo unnecessary surgical operations. Here, we describe an anisakiasis case that was correctly diagnosed clinically and treated successfully with conservative therapy, although the patient developed a 50-mm abscess but without percutaneous drainage.

## CASE REPORT

A 36-year-old man presented to our hospital with a complaint of severe abdominal pain. He had been well until the day before admission, when he had severe abdominal pain and several vomiting episodes with fever of 37.5°C. He had eaten sliced raw salmon 12 h before the onset. He denied diarrhea, and his past medical history was unremarkable. He did not smoke or drink. On physical examination, his abdomen showed diminished bowel sound and severe tenderness on the lower portion, with rebound tenderness (Blumberg’s sign) and no rigidity. The findings from the remainder of the physical examination were unremarkable. Laboratory examination revealed severe leukocytosis of 20 900/μl with 90.2% neutrophils and 1% eosinophils, and elevated levels of C-reactive protein (22.3 mg/dl), blood urea nitrogen (25 mg/dl) and creatinine (0.73 mg/dl). Other blood screening test results and the electrocardiogram were normal. Abdominal computed tomography (CT) revealed a small amount of ascites and enteritis without freeair.

The patient was admitted under the diagnosis of severe enteritis and placed under fasting status. Blood and stool samples were taken before the administration of 2 g/day cefmetazole (Sankyo Co., Ltd) intravenously (IV) in two divided doses. On day 3, the patient had fever with a body temperature of 39.6°C, and the antibiotic was switched from cefmetazole to 2 g/day imipenem and cilastatin (Sawai Pharmaceutical Co., Ltd) IV in four divided doses.

**Figure 1:**
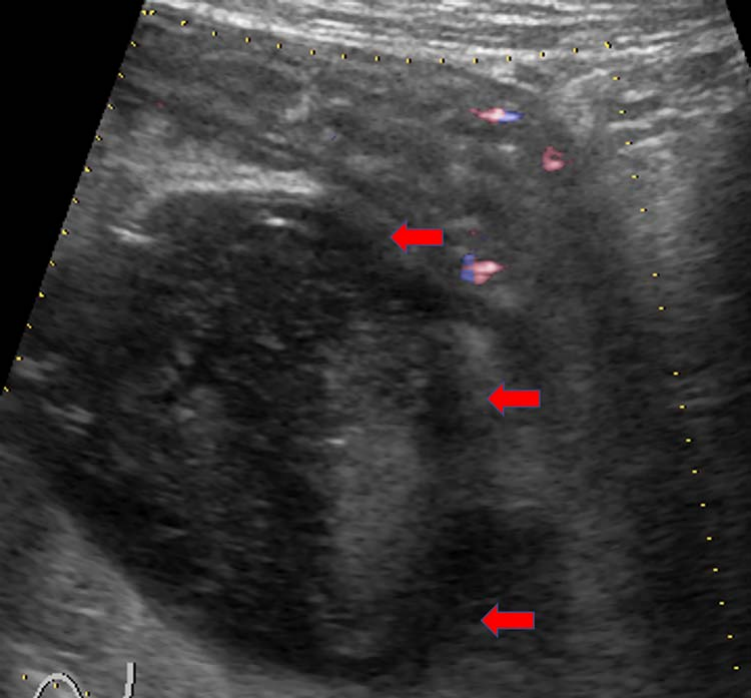
Abdominal echo image showing a 50-mm heterogeneous mass adjacent to the swollen ileum (red arrows).

**Figure 2:**
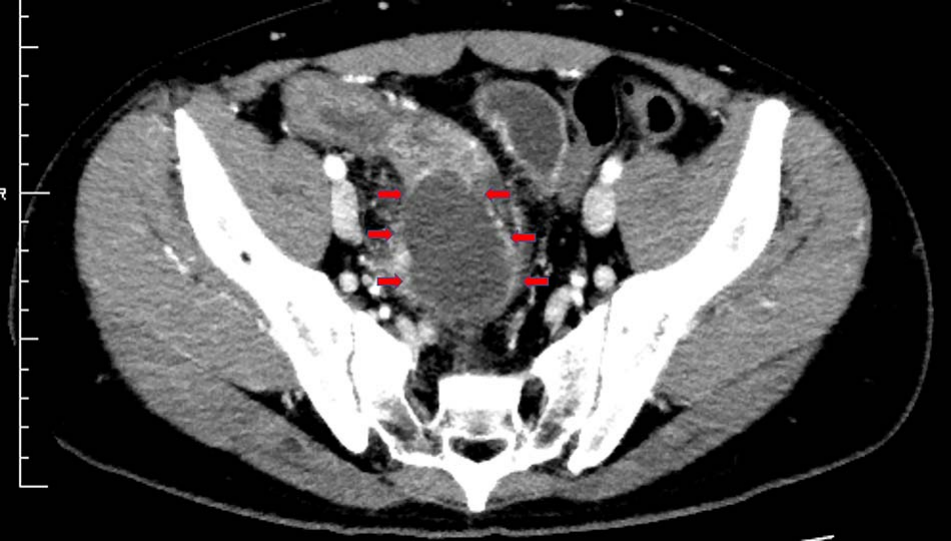
A contrast-enhanced computed tomography (CECT) scan showing a typical abscess finding of a 50-mm well-defined hypodense lesion with peripheral ring enhancing located next to the inflamed ileum (red arrows).

On day 7, the blood and stool cultures were negative, and the fever subsided to 37.3°C. Nonetheless, the abdominal pain and leukocytosis of 12 300/μl with 85.1% neutrophils persisted. Abdominal echo revealed a 50-mm heterogeneous mass adjacent to the swollen ileum (Fig. 1). A contrast-enhanced CT (CECT) revealed a typical abscess presenting as a 50-mm well-defined hypodense lesion with peripheral ring enhancement located next to the inflamed ileum (Fig. 2). Percutaneous drainage was impossible because the abscess was located in the deep back side of the pelvis. The treatment options were open surgery or conservative treatment with current imipenem and cilastatin IV without drainage. The patient chose conservative treatment under adequate given information, as the symptoms were subsiding day byday.

The test result for *Anisakis*-specific immunoglobulin A (IgA) on day 6 was negative (0.41 I/U; normal range <1.50 I/U), but that with paired sera on day 37 revealed an elevated value of 1.51 I/U. On day 15, the patient gradually resumed oral intake because his abdominal pain was gone. His white blood cell count became normal (7900/μl), and abdominal echo revealed that the abscess had shrunk to 20 mm in diameter. The IV antibiotics were discontinued on day 16. The patient was discharged on day 23 after the confirmation that increasing diet did not aggravate the symptom. The abscess shrunk to 10 mm on day 37.

## DISCUSSION

Anisakiasis can be confirmed by detecting the morphological characteristics of the whole worm on endoscopy [3, 5]. Different from gastric anisakiasis, intestinal anisakiasis is difficult to diagnose because the small intestine is an inaccessible zone for endoscopy. The patient’s history of eating sliced raw salmon made us suspect anisakiasis. The diagnosis was confirmed on the basis of *Anisakis*-specific IgA antibody level in the paired sera. The paired sera taken several weeks from the first test were effective for the diagnosis, with a reported sensitivity of ~70% if the test is performed early after onset [6–8].

A 50-mm abscess appeared without free air and pan-peritonitis, presumably because of the perforation in the mesentery side in this case. Although abscess should be treated with a combination of drainage and antibiotics, the patient was treated only with antibiotics owing to the location of the abscess precluding percutaneous drainage. Remarkably, even an abscess as large as 50 mm could be successfully treated without drainage in this case. The abscess shrunk to less than half its original size just after 1 week and to 10 mm after 1 month, presumably because the internal fistulation connected the abscess and the ileum.

Intestinal anisakiasis cannot be suspected even with detailed medical history taking along, as the symptoms are nonspecific; even CECT cannot detect it [6]. As a result, some patients with intestinal anisakiasis have undergone surgical exploration after diagnosis of acute abdomen or intestinal obstruction. As *Anisakis* can live in the human body for only several days, conservative treatment can be applicable widely [3, 6].
